# Curcuminoid Contents in Rhizomes of Some Zingiberaceous Plants Sold via Online Platforms: Influence of Species and Cultivation Location

**DOI:** 10.1155/2024/5929119

**Published:** 2024-06-11

**Authors:** Chaowalit Monton, Orawan Theanphong, Pathamaporn Pathompak, Jirapornchai Suksaeree, Natawat Chankana

**Affiliations:** ^1^ Drug and Herbal Product Research and Development Center College of Pharmacy Rangsit University, Pathum Thani 12000, Thailand; ^2^ Department of Pharmacognosy College of Pharmacy Rangsit University, Pathum Thani 12000, Thailand; ^3^ Department of Pharmaceutical Chemistry College of Pharmacy Rangsit University, Pathum Thani 12000, Thailand; ^4^ Sun Herb Thai Chinese Manufacturing College of Pharmacy Rangsit University, Pathum Thani 12000, Thailand

## Abstract

Zingiberaceous plants are versatile and find application in various fields, including food, medicine, and cosmetics. Recently, turmeric and other Zingiberaceous plants have become readily available through online platforms. However, the quality, specifically the curcuminoid content, has not been adequately assessed. In light of this issue, this study is aimed at analyzing the curcuminoid contents, including bisdemethoxycurcumin, demethoxycurcumin, and curcumin, using high-performance liquid chromatography. The analysis targets the rhizomes of *Zingiber montanum* (ZM), *Curcuma aromatica* (CA), *Curcuma wanenlueanga* (CW), *Curcuma zedoaria* (CZ), and sixteen *Curcuma longa* (CL) samples sold on online platforms. The influence of species and cultivation locations was evaluated, compared, and clustered. The results indicated that CL exhibited the highest curcuminoid contents, followed by CA, CZ, ZM, and CW, respectively. Curcumin was not detected in CW, while bisdemethoxycurcumin and demethoxycurcumin were absent in ZM. Cluster analysis revealed that CW was closely related to ZM, and CA was closely related to CZ, while CL was not closely related to either cluster. Among the sixteen CL samples, the most commonly found curcuminoids were curcumin, followed by bisdemethoxycurcumin and demethoxycurcumin, respectively. Three samples contained curcuminoid contents of less than 5%, failing to meet the standard level specified in the Thai Herbal Pharmacopoeia. Furthermore, ten samples had total curcuminoid contents higher than 10%, with three samples exceeding 15%. The top three samples with the highest total curcuminoid contents from different locations were as follows: Tha Yang District, Phetchaburi Province (17.02%); Phop Phra District, Tak Province (16.97%); and Pak Tho District, Ratchaburi Province (15.45%). Cluster analysis revealed that CL samples could be grouped into two major categories: low curcuminoid and high curcuminoid groups. This study offers valuable insights for consumers seeking high-quality rhizomes of Zingiberaceous plants with high curcuminoids, through online platforms. By focusing on the curcuminoid content, consumers can make informed decisions when purchasing Zingiberaceous plants online. This information not only aids in selecting superior quality rhizomes but also enhances the overall consumer experience by ensuring the potency and efficacy of the purchased products.

## 1. Introduction

The Zingiberaceae family, also known as the ginger family, is an important plant family consisting of over 1960 species distributed across tropical and subtropical regions around the world [1]. In Thailand, several reports have been published regarding the number of plant species belonging to the Zingiberaceae family. In 2008, 29 species were reported at Khao Nan and Khao Luang National Parks in Nakhon Si Thammarat Province [2]. Then, in 2014, 35 species were reported at Khao Luang National Park [3]. In 2016, 38 species were reported in Nam Nao National Park, spanning Chaiyaphum and Phetchabun Provinces [4]. In 2022, 67 species were reported in Bueng Kan Province [5]. In 2023, 33 species were reported in Mae Hong Son Province [6] and 155 species in Nakhon Nayok Province [1].

Curcuminoids—bisdemethoxycurcumin (BDMC), demethoxycurcumin (DMC), and especially curcumin (CUR)—have been proven to possess antioxidant, neuroprotective, antitumor, anti-inflammatory, antiacidogenic, radioprotective, and antiarthritis activities [7]. Several Zingiberaceous plants are recognized as curcuminoid-rich plants such as *Curcuma longa* L. [8–12], *Curcuma aromatica* Salisb. [9, 13–15], *Curcuma zedoaria* (Christm.) Roscoe [9, 16], and *Zingiber montanum* (J.Koenig) A.Dietr. [17–19]. *Curcuma wanenlueanga* Saensouk, Thomudtha & Boonma, a new species reported in 2021 [20], also contained yellow pigments which were possibly curcuminoid-contained plants. However, curcuminoid content was not previously reported for this species. Among several curcuminoids, CUR is an abundant compound that has been extensively studied. There was a moderate level of evidence indicating that turmeric/curcumin can be beneficial in alleviating pain and enhancing physical function in osteoarthritis. On the other hand, the evidence supporting its efficacy in addressing metabolic syndrome, inflammatory disorders, and other health issues was of lower quality. The data from systematic reviews suggested that both whole turmeric and formulations designed to enhance its bioavailability could potentially offer benefits for various health conditions. Furthermore, it was observed that turmeric (*C. longa*) is generally safe and well tolerated [21]. In Thailand, curcuminoids were launched in the market as modern medicine for the treatment of osteoarthritis. Furthermore, turmeric is a medicinal plant listed in the National List of Essential Medicines for the treatment of flatulence [22].

Zingiberaceous plants have been utilized in Thai traditional medicines for a long time, both as single herbs and in combination with other herbs in herbal formulas. Among them, *C. longa*, *C. zedoaria*, and *Z. montanum* have been listed in the monograph of the Thai Herbal Pharmacopoeia, indicating their importance and widespread use. Recently, turmeric, along with other Zingiberaceous plants cultivated in various locations across Thailand, has become readily available through online platforms. However, the quality, specifically the curcuminoid content, has not been adequately evaluated. The primary aim of this study was to analyze the curcuminoid contents within the rhizomes of various Zingiberaceous plants, including *Z. montanum*, *C. aromatica*, *C. wanenlueanga*, *C. zedoaria*, and sixteen samples of *C. longa* available on online platforms. Additionally, a cluster analysis was conducted to elucidate the similarities among different Zingiberaceous plant species and the various *C. longa* samples sourced from diverse locations across Thailand. This cluster analysis facilitated the discernment of patterns of similarity and variation in curcuminoid profiles, providing a deeper understanding of the chemical composition of these plants. By integrating cluster analysis into this investigation, the authors aimed to highlight the unique chemical profiles of each Zingiberaceous plant species and the variability present within *C. longa* samples from different geographical regions. The findings of this study are expected to offer valuable insights for consumers and researchers alike. Specifically, the identification of distinct curcuminoid profiles can serve as a practical guide for selecting high-quality rhizomes of Zingiberaceous plants, with a particular focus on turmeric rhizomes, when purchasing them from online platforms.

## 2. Materials and Methods

### 2.1. Materials

BDMC (batch no. PRF9010942, 99.38% purity), DMC (batch no. PRF9083121, 99.42% purity), and CUR (batch no. PRF10052344, 99.98% purity) standards were purchased from Chengdu Biopurify Phytochemicals Ltd., Sichuan, China. Acetonitrile (HPLC grade) was purchased from Fisher Chemical, Leicestershire, UK. Acetic acid (AR grade) was purchased from Carlo Erba Reagents GmbH, Milano, Italy.

### 2.2. Plant Materials

Four samples of fresh rhizomes from Zingiberaceous plants (*Z. montanum*, *C. aromatica*, *C. longa*, *C. wanenlueanga*, and *C. zedoaria*) were obtained from the same source, Chiang Dao District, Chiang Mai Province. Additionally, fresh *C. longa* rhizomes were harvested from various locations in Thailand via online platforms in July 2023. Therefore, this study included five Zingiberaceous species. A total of sixteen turmeric samples, four from northern Thailand, three from central Thailand, four from western Thailand, two from eastern Thailand, and three from southern Thailand, were included in this work. These samples were identified by Asst. Prof. Dr. Orawan Theanphong from the College of Pharmacy at Rangsit University to ensure the correct plant species. Voucher specimen numbers were assigned, and the samples were deposited at the Drug and Herbal Product Research and Development Center, College of Pharmacy, Rangsit University. The sample information is presented in Table 1. The samples were coded as ZM for *Z. montanum*, CA for *C. aromatica*, CW for *C. wanenlueanga*, CZ for *C. zedoaria*, and CL1 to CL16 for *C. longa*. They were cleaned with tap water, air-dried for 3 h, sliced to a thickness of approximately 2-3 mm, and further air-dried for 1 h. Subsequently, they were dried in a hot air circulating oven (RXH14-B, Changzhou Wangqun Pharmaceutical Machine Co., Ltd., Jiangsu, China) at 60°C for 5 h. The dried rhizomes were pulverized using a grinder (NT-500D, Nanotech, China) into a fine powder by pulsing, with a start time of 20 s and a stop time of 10 s, to avoid excessive heat during pulverization process. Then, they were sieved through a 60-mesh sieve and stored in a dry place until use.

### 2.3. Sample Preparation

The sample preparation procedure was adapted from the Thai Herbal Pharmacopoeia (THP) [23]. Each sample was accurately weighed to 10 mg into a test tube (*n* = 3), and 10 mL of methanol was added. The test tubes were then closed with caps and subjected to ultrasonication for 30 mins using an ultrasonication bath (Elmasonic Easy 300H, Elma Schmidbauer GmbH, Singen, Germany). Afterward, the samples were filtered through a 0.45 *μ*m pore size nylon syringe filter before being immediately analyzed by high-performance liquid chromatography (HPLC) to prevent the degradation of certain chemicals.

### 2.4. HPLC Condition

The HPLC analysis was slightly modified from the previous work [9, 11]. Curcuminoid content was analyzed using an HPLC instrument (Agilent 1260 Infinity, Agilent Technologies, California, USA). The separation was performed on the ACE Generix 5 C18 column (150 × 4.6 mm, i.d., 5 *μ*m) with temperature controlled at 30°C. The mobile phase consisted of acetonitrile and 1% acetic acid aqueous solution in the ratio of 50 : 50 at a flow rate of 1 mL/min. The injection volume was 10 *μ*L, and a signal was detected at 425 nm. Contents of each curcuminoid, BDMC, DMC, and CUR, were calculated from the calibration curves of each curcuminoid. The summation of the contents of each curcuminoid was reported as total curcuminoid content. Average value and standard deviation (SD) were reported. Furthermore, the Box-Whisker plot from the Tukey method was also produced.

### 2.5. Cluster Analysis

Cluster analysis was performed using IBM SPSS Statistics 22 (IBM, New York, USA). Hierarchical cluster analysis was applied to categorize the similarity of BDMC, DMC, and CUR contents in rhizomes of Zingiberaceous plants. Agglomeration schedule was a parameter used. The cluster method was Ward's method. The measuring interval was squared Euclidean distance. Furthermore, a dendrogram was also produced.

### 2.6. Statistical Analysis

Differences between two groups were analyzed using Student's *t*-test, while differences among multiple groups were analyzed using a one-way analysis of variance (one-way ANOVA) followed by LSD post hoc analysis in SPSS Statistics 22.0 (IBM, New York, USA). Data were considered significantly different when the *p* value was <0.05 at a 95% confidence interval.

## 3. Results and Discussion

### 3.1. Curcuminoid Contents of Different Zingiberaceous Plants

**Figure 1:**
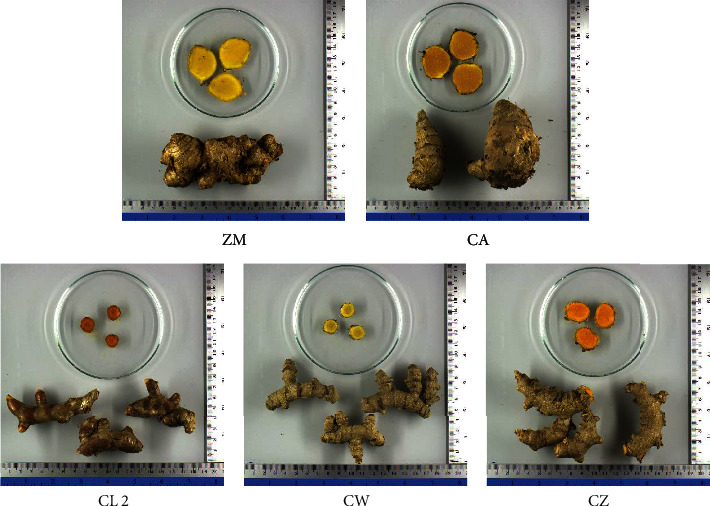
The fresh rhizomes of Zingiberaceous plants, *Z. montanum* (ZM), *C. aromatica* (CA), *C. longa* (CL 2), *C. wanenlueanga* (CW), and *C. zedoaria* (CZ), cultivated in Chiang Dao District, Chiang Mai Province, and obtained from online platforms.

**Figure 2:**
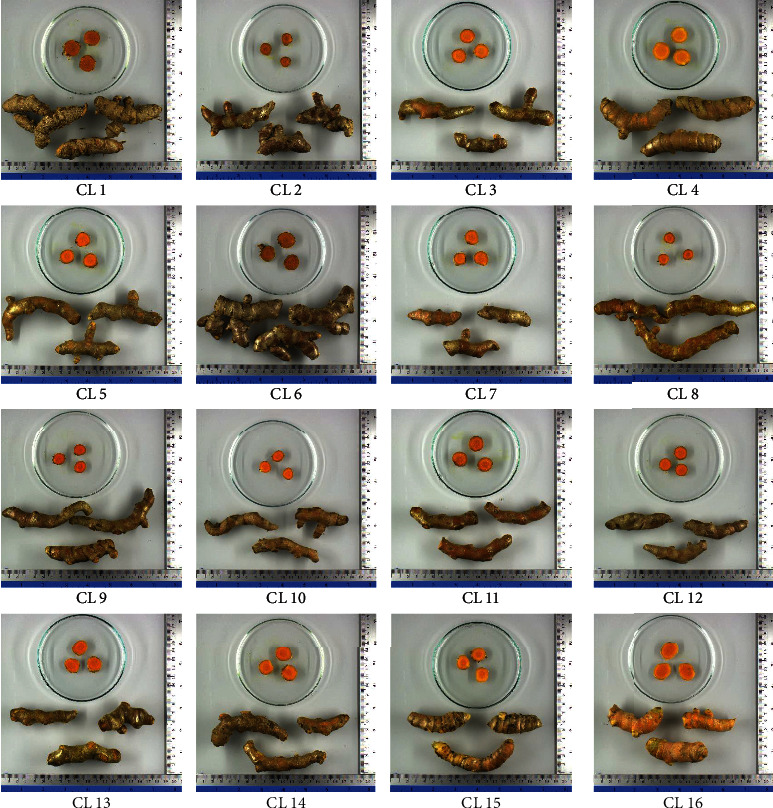
The fresh rhizomes of *C. longa* coded as CL 1 to CL 16 cultivated in various locations in Thailand and obtained from online platforms.

The fresh rhizomes of Zingiberaceous plants used in this study are depicted in Figures 1 and 2.  Figure 1 displays various sizes and colors of the fresh rhizomes from these plants. Among them, ZM and CA exhibited larger rhizomes compared to the other plants. CL rhizomes were dark orange, while CZ and CA had orange ones, and ZM and CW had yellow rhizomes. In the case of the CL samples, Figure 2 illustrates varying sizes and intensities of orange color.

**Figure 3:**
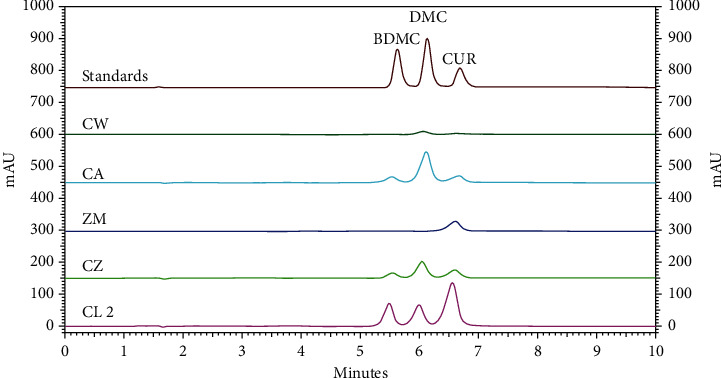
The HPLC chromatograms of curcuminoid standards (BDMC, DMC, and CUR, each at 12.5 *μ*g/mL) and extracts of *C. wanenlueanga* (CW), *C. aromatica* (CA), *Z. montanum* (ZM), *C. zedoaria* (CZ), and *C. longa* (CL 2).

**Figure 4:**
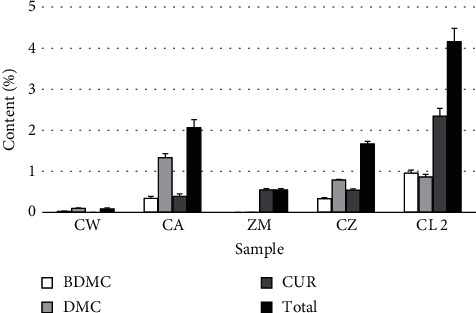
Contents of individual curcuminoid, BDMC, DMC, and CUR, and total curcuminoid in the rhizomes of Zingiberaceous plants, *C. wanenlueanga* (CW), *C. aromatica* (CA), *Z. montanum* (ZM), *C. zedoaria* (CZ), and *C. longa* (CL 2), cultivated in Chiang Dao District, Chiang Mai Province, and obtained from online platforms.

The HPLC chromatograms of curcuminoid standards and extracts of Zingiberaceous plants are shown in Figure 3. The contents of individual curcuminoids and total curcuminoids in the rhizomes of various Zingiberaceous plants cultivated in the same location, Chiang Dao District, Chiang Mai Province, are presented in Figure 4. Among these, CW and CL had the lowest and the highest curcuminoid content, respectively. CA, CZ, and CL contained three curcuminoids, similar to the previous study [9]. DMC was the most abundant curcuminoid in CW, CA, and CZ, while CUR was the predominant curcuminoid in ZM and CL. This study revealed that CW lacked CUR and ZM lacked both BDMC and DMC. However, there is limited prior publication on the chemical profile of CW, as it is a newly reported species, making it challenging to compare our results with existing data. Regarding ZM, the authors observed a low curcuminoid content, and BDMC and DMC were not detected similar to the previous publication [24]. However, these compounds could be found in petroleum ether and ethyl acetate extracts isolated by fractionation [19] and ethanolic extract obtained from maceration [25]. The authors mentioned that the present work used methanol as the extraction solvent during sample preparation for HPLC analysis, as specified in the THP [23]. The official method was simple, and the solvent used was safer than in earlier work [19]. Furthermore, this work utilized ultrasonication for the extraction of curcuminoids from the samples, which can accelerate the extraction process. Therefore, it can shorten the sample preparation process compared to the maceration technique [26].

### 3.2. Cluster Analysis of Different Zingiberaceous Plants

Employing hierarchical cluster analysis with Ward's method and squared Euclidean distance enhances the interpretability of results in several ways. It enables visualization of relationships between samples through dendrogram plots, highlighting the hierarchical structure of similarity within the dataset. This visualization aids in identifying clusters of samples with similar curcuminoid profiles, providing insights into potential patterns or groupings that may correspond to specific characteristics [27].

**Figure 5:**
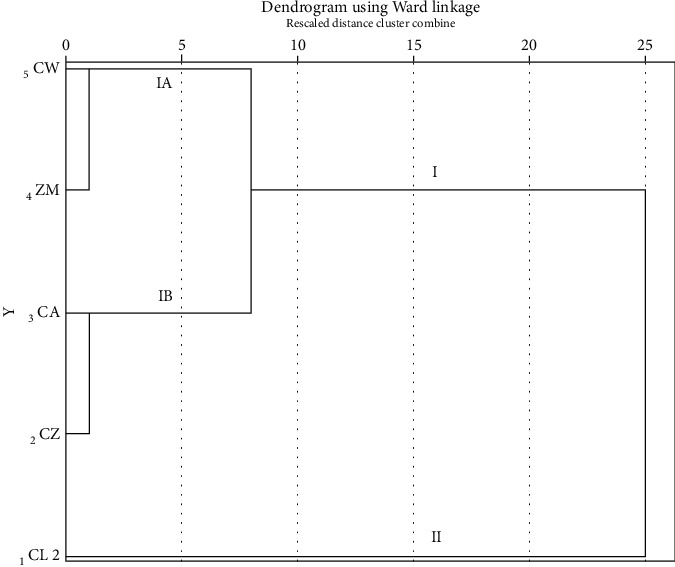
Dendrogram of hierarchical cluster analysis based on BDMC, DMC, and CUR contents in the rhizomes of Zingiberaceous plants, *Z. montanum* (ZM), *C. aromatica* (CA), *C. longa* (CL 2), *C. wanenlueanga* (CW), and *C. zedoaria* (CZ), cultivated in Chiang Dao District, Chiang Mai Province, and obtained from online platforms.

The dendrogram resulting from hierarchical cluster analysis, which is based on the contents of BDMC, DMC, and CUR in the rhizomes of Zingiberaceous plants, is depicted in Figure 5. The Zingiberaceous plants can be divided into two distinct groups: non-*C. longa* (group I) and *C. longa* (group II). This classification is primarily driven by the high curcuminoid content present in CL, particularly in terms of CUR content. Within group I, a further subdivision can be observed into two subgroups: group IA, consisting of CW and ZM, characterized by a low curcuminoid content, and group IB, comprising CA and CZ, which exhibit a medium curcuminoid content, notably with high DMC levels.

### 3.3. Curcuminoid Contents of *C. longa* Rhizomes from Various Cultivation Locations

**Figure 6:**
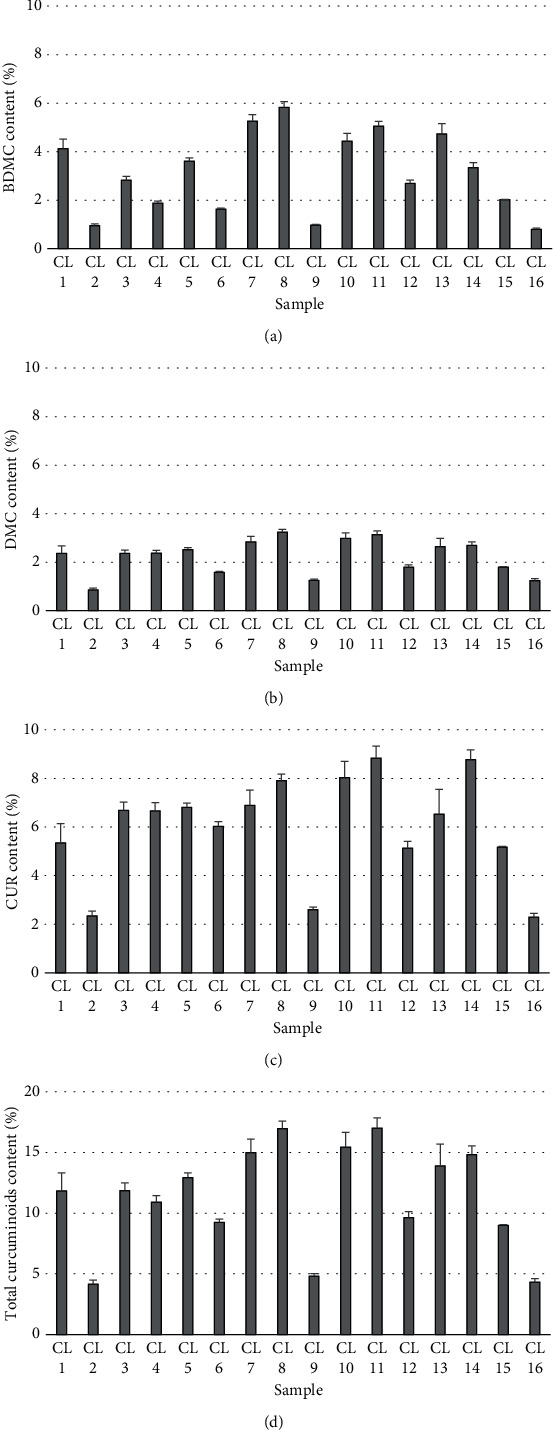
Contents of (a) BDMC, (b) DMC, (c) CUR, and (d) total curcuminoid in the rhizomes of *C. longa* cultivated in various locations in Thailand and obtained from online platforms.

The influence of the cultivation location of CL on curcuminoid contents was assessed by procuring CL rhizomes from various cultivation locations through online platforms. The individual curcuminoids, including BDMC, DMC, and CUR, as well as the total curcuminoid content in the rhizomes of CL cultivated in different locations, are depicted in Figure 6. The average value of BDMC obtained in this study was 3.13%, with a range of 0.80% to 5.83%. The minimum and maximum BDMC contents were found in CL 16 and CL 8, respectively. The average value of DMC was 2.23%, ranging from 0.86% to 3.24%. The minimum and maximum DMC contents were observed in CL 2 and CL 8, respectively. The average value of CUR was 6.00%, with a range of 2.29% to 8.83%. The minimum and maximum CUR contents were identified in CL 16 and CL 11, respectively. The average value of the total curcuminoid was 11.37%, ranging from 4.16% to 17.02%. The minimum and maximum total curcuminoid contents were found in CL 2 and CL 11, respectively. These results indicate that the cultivation location affects the curcuminoid content of CL rhizomes, and this can be influenced by geographic factors (such as altitude), climatic factors (such as temperature, rainfall, and season of harvesting), edaphic factors (such as soil type and compositions), etc. [28–31].

**Figure 7:**
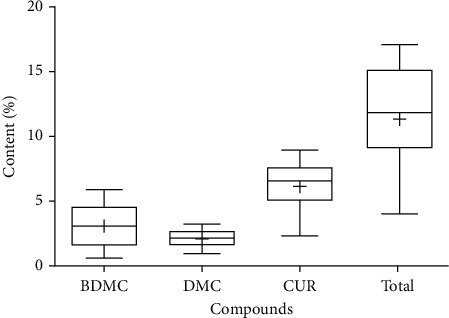
Box-Whisker plot represented BDMC, DMC, CUR, and total curcuminoid contents of *C. longa* rhizomes cultivated in various locations in Thailand and obtained from online platforms.

The THP specifies that qualified turmeric powder should contain a total curcuminoid content of not less than 5% [23]. This study revealed that out of the 16 samples examined, 13 met the THP standards. Furthermore, ten samples exhibited total curcuminoid contents exceeding 10%, with three of them exceeding 15%. The top three samples with the highest total curcuminoid contents, originating from different locations, were as follows: CL 11 from Tha Yang District, Phetchaburi Province (17.02%); CL 8 from Phop Phra District, Tak Province (16.97%); and CL 10 from Pak Tho District, Ratchaburi Province (15.45%). Formerly, the curcuminoid content in turmeric rhizomes varied widely, ranging from 0.005% to 12.39%. This variability can be detailed as follows: 0.005-0.54% [32], 0.76% [33], 2.65% [34], 2.66% [35], 1.30-4.79% [31], 7.00-8.00% [8], 8.82% [36], and 0.46-12.39% [37]. Therefore, the present study highlights that the CL samples used in this work exhibit a high curcuminoid content. Furthermore, a Box-Whisker plot was used to represent the BDMC, DMC, CUR, and total curcuminoid contents of CL rhizomes cultivated in various locations in Thailand and obtained from online platforms as shown in Figure 7. The plot revealed that the CL samples used in this study had CUR as the most abundant compound, followed by BDMC and DMC, respectively.

The study delves into the impact of cultivation location on the curcuminoid content of CL rhizomes, comparing the findings with existing literature and highlighting novel insights. The results align with prior research indicating significant effects of geographical and environmental factors on curcuminoid levels in CL rhizomes. The observed variability across cultivation locations is consistent with established knowledge regarding how factors such as altitude, climate, and soil composition influence curcuminoid biosynthesis. However, the study also reveals discrepancies compared to some previous findings. While past reports have documented a wide range of curcuminoid content in turmeric rhizomes, the study consistently found relatively high curcuminoid levels across the tested samples. This variance suggests potential differences in CL cultivars, cultivation methods, or regional factors requiring further exploration.

Geographic factors play a crucial role in shaping the curcuminoid profiles of CL rhizomes. Altitude, for instance, affects the microclimate and sunlight exposure, which in turn influence the synthesis of curcuminoids. Climatic conditions, including temperature, rainfall, and seasonal variations, significantly impact curcuminoid content. Optimal temperature ranges and adequate rainfall patterns are known to promote the growth of CL and enhance curcuminoid accumulation. Seasonal variations, especially during the harvesting period, can influence the maturity and quality of rhizomes, affecting curcuminoid concentrations. Furthermore, soil type and composition (edaphic factors) also contribute to variations in curcuminoid profiles. Different soil properties such as pH, nutrient availability, organic matter content, and drainage can influence the uptake of nutrients and secondary metabolites by plants, including curcuminoids [29, 38–41]. Therefore, the interplay of these geographic, climatic, and edaphic factors results in the observed variations in curcuminoid content across different cultivation locations. In addition, genetic variation [42–44], cultivation and harvesting practices [45, 46], and processing and storage [10, 11, 47] can affect curcuminoid content. Understanding and accounting for these factors are essential for elucidating the complex mechanisms underlying curcuminoid biosynthesis and for optimizing turmeric cultivation practices to enhance curcuminoid yield and quality.

### 3.4. Cluster Analysis of *C. longa* Rhizomes from Various Cultivation Locations

**Figure 8:**
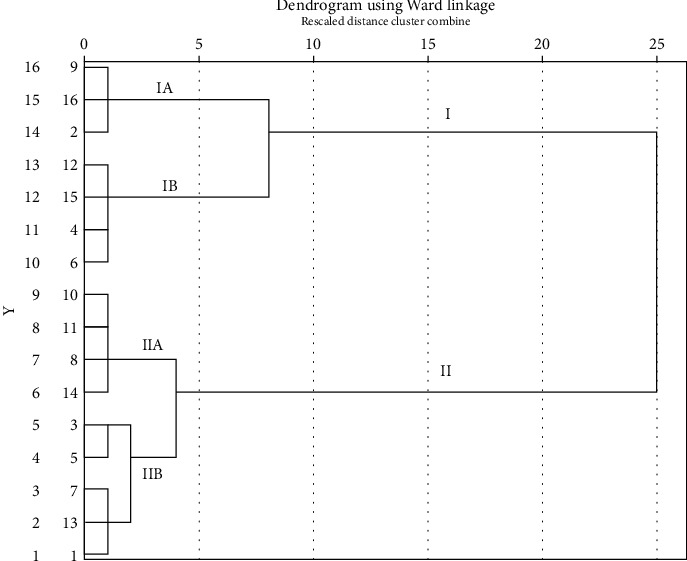
Dendrogram of hierarchical cluster analysis based on BDMC, DMC, and CUR contents of *C. longa* cultivated in various locations in Thailand and obtained from online platforms.

The dendrogram of the hierarchical cluster analysis based on BDMC, DMC, and CUR contents of CL cultivated in various locations in Thailand is presented in Figure 8. The CL samples used in this study can be grouped into two main categories: group I, the low curcuminoid group, and group II, the high curcuminoid group. A statistical analysis of BDMC, DMC, and CUR contents between these two groups revealed that group II had significantly higher BDMC, DMC, and CUR contents than group I, with *p* values of <0.0001, <0.0001, and 0.001, respectively. Within group I, it was further categorized into two subgroups: groups IA and IB. Group IA (including CL 2, CL 9, and CL 16) exhibited low BDMC, DMC, and CUR contents, while group IB (comprising CL 4, CL 6, CL 12, and CL 15) had significantly higher BDMC, DMC, and CUR contents in comparison to group IA, with *p* values of 0.045, 0.002, and <0.0001, respectively. Remarkably, group IA had a total curcuminoid content of less than 5%, indicating that this subgroup did not meet the standards of THP [23]. According to group II, it can be divided into two subgroups as well: groups IIA and IIB. Group IIA (including CL 8, CL 10, CL 11, and CL 14) exhibited elevated levels of BDMC, DMC, and CUR. Notably, group IIA demonstrated significantly higher levels of DMC and CUR compared to group IIB, although there was no significant difference in BDMC levels between the two groups. The *p* values for BDMC, DMC, and CUR between these two groups were 0.425, 0.016, and 0.002, respectively. Group IIB can be further categorized into two subgroups. The group consisting of CL 3 and CL 5 exhibited lower levels of BDMC but comparable DMC and CUR contents when compared to the group comprising CL 1, CL 7, and CL 13. However, there was no significant difference between these two subgroups, with *p* values of 0.063, 0.405, and 0.482, respectively.

## 4. Conclusions

The advent of online platforms has indeed increased the accessibility of turmeric and other Zingiberaceous plants to consumers. However, the study has brought to light a critical issue concerning the quality of turmeric and other Zingiberaceous products, specifically in terms of their curcuminoid content. This research is aimed at bridging this gap by conducting a comprehensive analysis of curcuminoid levels in turmeric and other Zingiberaceous rhizomes sold online, originating from various regions across Thailand. Cluster analysis of curcuminoid content in different Zingiberaceous species categorized them into CL and non-CL groups. Remarkably, among the CL samples, 13 out of 16 met the total curcuminoid content standards set by the THP. Turmeric samples from Tha Yang (Phetchaburi), Phop Phra (Tak), and Pak Tho (Ratchaburi) exhibited the highest total curcuminoid content. Cluster analysis revealed that CL samples could be grouped into two major categories: low curcuminoid and high curcuminoid groups. In summary, this study serves as a valuable resource for consumers seeking high-quality turmeric and other Zingiberaceous rhizomes through online platforms, especially those interested in products with elevated total curcuminoid contents. By shedding light on the variations in curcuminoid levels among different sources, this research contributes to informed decision-making and encourages the procurement of turmeric and other Zingiberaceous products that offer optimal health benefits and therapeutic potential.

## Figures and Tables

**Table 1:** Sample code, scientific name, voucher specimen number, and source of rhizomes of turmeric and other Zingiberaceous plants used in this work.

Sample code	Scientific name	Voucher specimen no.	Part of Thailand^∗^	Source	
				District	Province
ZM	*Z. montanum*	TMRC 022	Northern	Chiang Dao	Chiang Mai
CA	*C. aromatica*	TMRC 021	Northern	Chiang Dao	Chiang Mai
CW	*C. wanenlueanga*	TMRC 020	Northern	Chiang Dao	Chiang Mai
CZ	*C. zedoaria*	TMRC 023	Northern	Chiang Dao	Chiang Mai
CL 1	*C. longa*	TMRC 004	Northern	Wiang Pa Pao	Chiang Rai
CL 2	*C. longa*	TMRC 005	Northern	Chiang Dao	Chiang Mai
CL 3	*C. longa*	TMRC 006	Northern	Rong Kwang	Phrae
CL 4	*C. longa*	TMRC 007	Northern	Mueang	Lampang
CL 5	*C. longa*	TMRC 008	Central	Si Satchanalai	Sukhothai
CL 6	*C. longa*	TMRC 009	Central	Phatthana Nikhom	Lop Buri
CL 7	*C. longa*	TMRC 010	Central	Bang Kruai	Nonthaburi
CL 8	*C. longa*	TMRC 011	Western	Phop Phra	Tak
CL 9	*C. longa*	TMRC 012	Western	Tha Song Yang	Tak
CL 10	*C. longa*	TMRC 013	Western	Pak Tho	Ratchaburi
CL 11	*C. longa*	TMRC 014	Western	Tha Yang	Phetchaburi
CL 12	*C. longa*	TMRC 015	Eastern	Phanat Nikhom	Chon Buri
CL 13	*C. longa*	TMRC 016	Eastern	Na Yai Am	Chanthaburi
CL 14	*C. longa*	TMRC 017	Southern	Ban Na San	Surat Thani
CL 15	*C. longa*	TMRC 018	Southern	Cha-uat	Nakhon Si Thammarat
CL 16	*C. longa*	TMRC 019	Southern	Mueang	Phatthalung

^∗^Based on geography.

## Data Availability

The data used to support the findings of this study are available from the corresponding author upon request.
